# Systematic dissection of the mechanisms underlying progesterone receptor downregulation in endometrial cancer

**DOI:** 10.18632/oncotarget.2392

**Published:** 2014-09-03

**Authors:** Shujie Yang, Yichen Jia, Xiaoyue Liu, Christopher Winters, Xinjun Wang, Yuping Zhang, Eric J. Devor, Adriann M. Hovey, Henry D. Reyes, Xue Xiao, Yang Xu, Donghai Dai, Xiangbing Meng, Kristina W. Thiel, Frederick E. Domann, Kimberly K. Leslie

**Affiliations:** ^1^ Department of Obstetrics and Gynecology, University of Iowa, IA, 52242, USA; ^2^ The Interdisciplinary Graduate Program in Informatics, University of Iowa, IA, 52242, USA; ^3^ Free Radical and Radiation Biology Program, Department of Radiation Oncology, University of Iowa, IA, 52242, USA; ^4^ Carver College of Medicine and Holden Comprehensive Cancer Center, University of Iowa, IA, 52242, USA

**Keywords:** Progesterone receptor, progestin, epigenetic regulation, DNA methylation, histone deacetylase inhibitor

## Abstract

Progesterone, acting through its receptor, PR (progesterone receptor), is the natural inhibitor of uterine endometrial carcinogenesis by inducing differentiation. PR is downregulated in more advanced cases of endometrial cancer, thereby limiting the effectiveness of hormonal therapy. Our objective was to understand and reverse the mechanisms underlying loss of PR expression in order to improve therapeutic outcomes. Using endometrial cancer cell lines and data from The Cancer Genome Atlas, our findings demonstrate that PR expression is downregulated at four distinct levels. In well-differentiated cancers, ligand-induced receptor activation and downregulation are intact. miRNAs mediate fine tuning of PR levels. As differentiation is lost, PR silencing is primarily at the epigenetic level. Initially, recruitment of the polycomb repressor complex 2 to the PR promoter suppresses transcription. Subsequently, DNA methylation prevents PR expression. Appropriate epigenetic modulators reverse these mechanisms. These data provide a rationale for combining epigenetic modulators with progestins as a therapeutic strategy for endometrial cancer.

Significance: Traditional hormonal therapy for women with endometrial cancer can be molecularly enhanced by combining progestins with epigenetic modulators, thereby increasing progesterone receptor expression and significantly improving treatment efficacy.

## INTRODUCTION

Endometrial cancer is the most common gynecologic malignancy. Incidence and associated morbidity and mortality are rising, with an estimated 49,560 new cases and 8,190 deaths in 2013 [1]. The uterine endometrium is exquisitely sensitive to steroid hormones. Estrogen drives proliferation, while progesterone acts through progesterone receptors (PR: PRA, PRB and PRC) to counteract these effects by inducing differentiation, promoting apoptosis, and inhibiting invasion [2]. Therefore, progesterone is a powerful tumor suppressor in the endometrium, a function which has long been exploited therapeutically in progestin-based hormonal therapy for endometrial hyperplasia and carcinoma. Clinical studies have suggested that the efficacy of progestin therapy is high in endometrial hyperplasia, moderate in primary endometrial adenocarcinoma, but low in advanced and recurrent disease. For example, in advanced disease, only 15–33% of patients respond to progestin, and the therapeutic benefit generally lasts for only a short time [3, 4]. This trend of decreasing response rates with disease progression is thought to be linked to the loss of PR [5, 6]. As progestin therapy correlates with hormone receptors, maintaining or enhancing PR expression is an important goal with the potential to significantly improve clinical outcomes.

Data from The Cancer Genome Atlas (TCGA) generally confirm our traditional pathologic understanding of endometrial cancer, broadly referred to as Type I (70–80% incidence) and Type II (15–20% incidence) [7]. Type I tumors comprise the “low copy number” TCGA category which frequently harbor PTEN mutations, are associated with better differentiation and express higher levels of steroid hormone receptors including PR. By contrast, Type II tumors, which are most often serous or serous-like but also include some poorly differentiated endometrioid cases, comprise the “high copy number” TCGA category characterized by p53 mutations and loss of PR [2]. High risk cases from both categories require up front adjuvant treatment after initial hysterectomy, and patients with recurrent disease also benefit from second line therapy. In comparison to chemotherapy, hormonal therapy represents a safe and potentially effective treatment strategy for cancers that are hormonally dependent, defined by high expression of receptors. However, in advanced endometrial cancer PR expression is lost, thus limiting the usefulness of hormonal therapy as it is currently administered [8]. What is needed is to optimize hormonal therapy and re-instate tumor sensitivity by enhancing the expression of receptors, which we term “molecularly enhanced progestin-based therapy.”

Several different mechanisms have been reported which partially explain the decreased expression of PR in endometrial cancers, including ligand-mediated downregulation, miRNA-mediated translational suppression, and epigenetic factors which repress PR expression [2]. However, there is as yet no systematic study to understand the precise relationship between the reported mechanisms, how each mechanism links to different types of endometrial cancer, and what opportunities exist to reverse PR loss. The goals of our study were to answer these questions and to provide a strong rationale for the use of molecularly enhanced progestin-based therapy.

## RESULTS

### PR expression is downregulated in endometrial tumors

**Figure 1:**
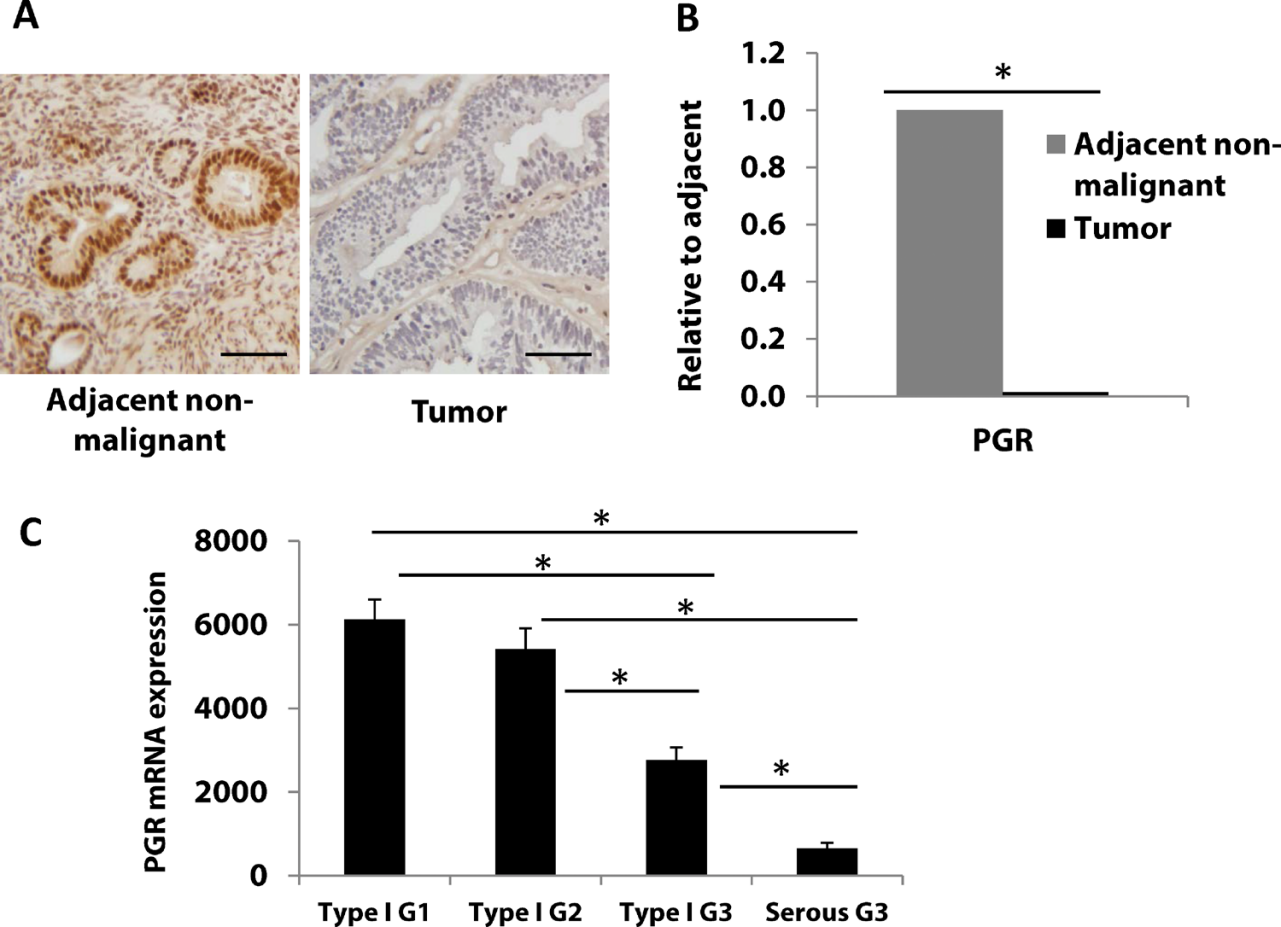
Progesterone receptor expression is frequently downregulated with progression of endometrial cancer **A, B** PR protein **(A)**, and *PGR* mRNA **(B)**, expression was measured in endometrial tumors (n=5) and matched adjacent non-malignant tissue (n=5) by immunostaining and real-time PCR, respectively. Scale bar = 50 μm. Student's t-test was used for comparisons of two groups. **(C)** *PGR* mRNA expression was analyzed in 361 endometrial cancer patient tumors from TCGA database. Patients were divided into four groups: endometrioid type I grade 1 (G1, n=84), grade 2 (G2, n=100), grade 3 (G3, n=115) and serous Type II grade 3 (G3, n=62). Error bars, SEM. Statistical analysis was conducted using one way ANOVA with significance level set at α=0.05. All pairwise multiple comparisons were performed using Holm-Sidak method with Bonferroni correction. The results showed that all individual groups are significantly different from each other (p< 0.001) except between Type I G1 and Type I G2 (p=0.209).

PR expression has been studied previously and has been found to decrease during endometrial cancer progression [4]. In this study, we confirmed this finding by studying tissues collected at our institution and also by analyzing data from The Cancer Genome Atlas (TCGA). Five de-identified early stage and grade endometrial tumor specimens, including adjacent non-malignant tissue, were evaluated for PR expression by immunohistochemistry (IHC). PR protein expression, as determined by the percent of stained cells multiplied by the staining intensity (1+, 2+ or 3+), was decreased in endometrial tumor lesions when compared to adjacent non-malignant tissue (Fig. 1A). Next, we investigated the PR mRNA (*PGR*) expression by q-PCR. As shown in Fig. 1B, *PGR* mRNA expression decreased significantly consistent with protein levels in the tumors compared to the non-malignant surrounding tissue (P<0.05 by student's t-test)

To further support the alteration of PR expression in an expanded sample size, we turned to the endometrial cancer TCGA database. In a previous report from the TCGA research network which assessed 333 endometrial tumors, high grade cases consistently expressed significantly less PR compared to low grade cases at both the mRNA and protein levels [7]. We further evaluated PR expression and correlated it with tumor grade in an expanded number of patients from the TCGA dataset. Fig. 1C shows that from 361 endometrial tumors, *PGR* mRNA expression decreased significantly from endometrioid endometrial cancers to more aggressive serous tumors. Among cases in the endometrioid tumor group, *PGR* expression was also found to be downregulated in grade 3 vs. grade 1 tumors (P<0.05 by one-way ANOVA followed by the Holm-Sidak method for pairwise comparisons). These data are consistent with data in Fig. 1A, B and previous observations that PR is lost in advanced endometrial cancer [9]. Next, we investigated the mechanisms underlying this finding.

### Ligand-dependent PR activation and downregulation

**Figure 2:**
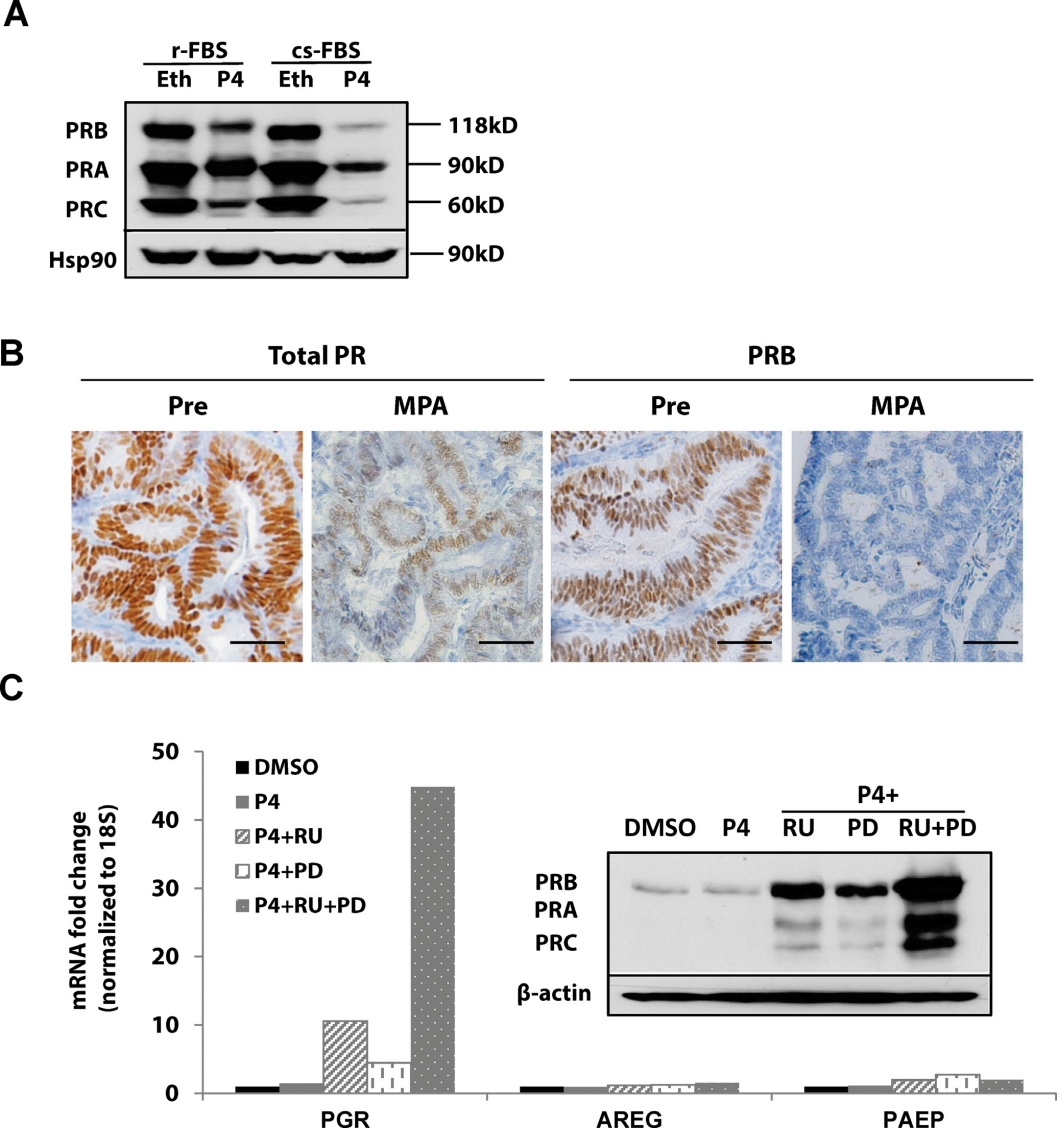
Ligand-dependent PR downregulation **(A)** Western blotting: T47D breast cancer cells were grown in DMEM media supplemented with regular fetal bovine serum (r-FBS) or charcoal-stripped serum (cs-FBS), followed by treatment with ethanol (vehicle control) or 100 nM progesterone (P4) for 24h. PR protein was detected using specific PR antibodies, and HSP90 serves as a loading control. **(B)** Immunohistochemistry: endometrial tumor specimens were collected before or 21 days after MPA treatment (400 mg, intramuscularly) and total PR and PRB expression was assessed by immunohistochemistry. Scale bar = 50 μm. **(C)** q-PCR and Western blotting: ECC1 cells were treated with DMSO (vehicle control), 100 nM P4, 100 nM P4 and 1μM PR antagonist RU486 (RU), 100 nM P4+ 1 μM MAPK inhibitor PD0325901(PD) or the combination of P4+RU+PD for 24h. mRNA expression of *PGR*, *AREG* and *PAEP* was measured by q-PCR, normalized to 18S, and data displayed as fold-change relative to DMSO control. Comparisons of normalized expression values (ΔCt) employed the conventional ΔΔCt fold change method. The insert is PR protein expression after the same treatment; β-actin, loading control.

Ligand-induced receptor activation and downregulation is a well-known phenomenon [10–12]. Progesterone-dependent PR activation and downregulation has been documented in both breast and endometrial cancer cells where phosphorylation of PR both activates the receptor and signals its ubiquitination and degradation by the proteasome [12, 13]. To further understand this mechanism of PR downregulation, we initially employed T47D breast cancer cells as a model. As shown in Fig 2A, three PR isoforms (PRB, PRA and PRC) were detected by immunoblotting and found to be decreased when cells were treated with progesterone (Fig. 2A). Figure 2B is a representative immunohistochemical analysis from pre- and post-treatment endometrial biopsies from a patient with stage II, grade 2 endometrial cancer treated with medroxyprogesterone acetate (MPA) prior to hysterectomy. The observed loss of PR is consistent with receptor activation followed by histologic evidence of response to progestin, which is accompanied by the ultimate downregulation of PR. We next confirmed that ligand-dependent PR downregulation occurs in endometrial cancer cells and used these models to reverse this mechanism, which involves ligand induced MAPK-mediated PR phosphorylation and activation. RU486, a PR antagonist, and PD0325901, a MAPK inhibitor, were employed in these studies. Hormonally responsive and well-differentiated ECC1 endometrial cancer cells were treated with progesterone +/− the inhibitors. Treatment with either RU486 or PD0325901 alone increased PR protein expression (Figs. S1 and 2C); this is consistent with the impact of these agents as antagonists of ligand-activated PR phosphorylation and degradation. The combination of RU486 and PD0325901 further magnified this effect. We next examined mRNA levels of *PGR* as well as the expression of two classic PR target genes, amphiregulin (*AREG*) and progesterone-associated endometrial protein (*PAEP*), also known as glycodelin. Whereas *PGR* levels were induced by 40-fold in cells treated with both drugs (Fig. 2C), *AREG* and *PAEP* mRNA levels were low, indicating that the preserved PR is transcriptionally inactive when cells are treated with RU486 or PD0325901. These data confirm that ligand-dependent PR downregulation is part of the normal cycle of PR activity in the endometrium and signifies cells which actively express PR-induced genes. However, with ongoing exposure to progestin therapy, PR is transcriptionally silenced, as discussed below.

### PR promoter methylation is one mechanism for PR repression in endometrial cancer

**Figure 3:**
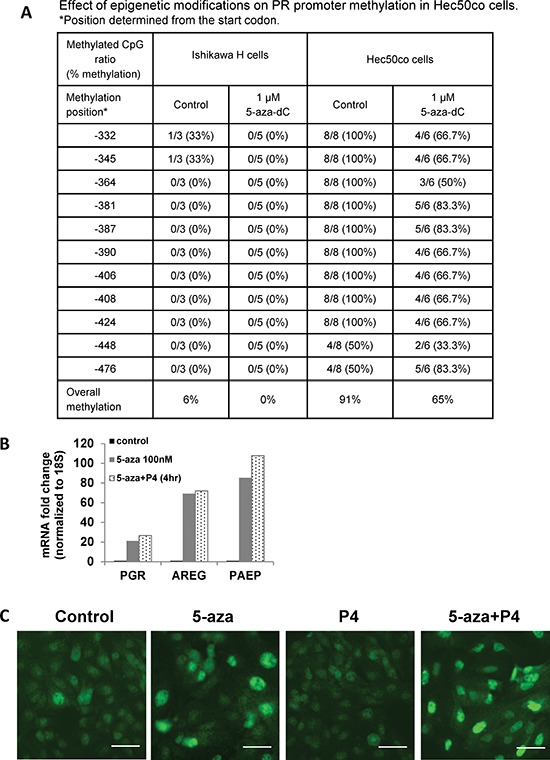
*PGR* promoter methylation represses PR expression in serous endometrial cancer cells **(A)** Bisulfite sequencing. *PGR* promoter methylation was quantified in Ishikawa H and Hec50co cells before or after hypomethylating agent 5-aza-decitabine (5-aza-dC) treatment. **(B)** q-PCR analysis. Hec50co cells were treated with DMSO (vehicle control), 100 nM 5-aza-dC for 5 days with fresh 5-aza-dC added every other day, 5-aza-dC for 5 days +100 nM P4 for 4h. mRNA expression of *PGR, AREG* and *PAEP* was measured by q-PCR and normalized to 18S, and data are displayed as fold-change relative to DMSO control. Comparisons of normalized expression values (ΔCt) employed the conventional ΔΔCt fold change method. **(C)** Representative immunofluorescent image showing PR expression in Hec50co cells treated with DMSO control, 5 days of 100 nM 5-aza-dC, 100 nM P4, or both. Scale bar = 50 μm.

Promoter methylation of tumor suppressors is common in cancer [14, 15]. We hypothesized that PR silencing occurs at this level in some endometrial cancer cells and tumors. *PGR* promoter methylation was assessed in a panel of eight endometrial cancer cell lines using bisulfite sequencing. Five endometrial cancer cell lines (KLE, AN3CA, SKUT1B, ECC1 and Ishikawa H cells) had little CpG methylation, but three endometrial cancer cell lines (RL95, Hec1A and Hec50co) had high CpG methylation on the *PGR* promoter (Fig. S2A). In cell lines with significant *PGR* promoter methylation, expression of *PGR* mRNA was low (Fig. S2B). To further study DNA methylation, we used previously reported cell models of Type I moderately differentiated endometrial cancer (Ishikawa H) and Type II aggressive serous endometrial cancer (Hec50co) [16]. Strikingly, 91% of the *PGR* promoter was methylated in the Type II Hec50co cells compared to only 6% in Type I Ishikawa H cells (Fig. 3A). Treatment with the hypomethylating agent 5-aza-decitabine (5-aza-dC) partially reversed *PGR* promoter methylation in Hec50co cells (from 91% to 65%). We next assessed mRNA levels of *PGR*, *AREG* and *PAEP*. Consistent with promoter demethylation, treatment of Hec50co cells with 5-aza-dC increased mRNA levels of *PGR* by 20-fold, *AREG* by 60-fold and *PAEP* by 80-fold (Fig. 3B), but not the oncogene *Myc* which is not considered to be regulated by methylation (Fig. S2C). As expected, 5-aza-dC treatment also increased *PGR* expression in RL95 cells where CpG methylation of the *PGR* promoter is robust (Fig. S2D). To validate that the increased expression of *AREG* and *PAEP* is PR-dependent, progesterone was added to the cell media to activate PR transcriptional activity. The addition of progesterone to 5-aza-dC-treated Hec50co cells further increased *AREG* and *PAEP* expression, presumably as a result of progesterone-mediated PR transcriptional activation. These data demonstrate that the restored PR is functional. Analysis of PR expression in Hec50co cells by immunostaining with a fluorescent antibody [17] revealed that 5-aza-dC increased nuclear PR expression, while the combination of progesterone and 5-aza-dC further increased nuclear PR levels (Fig. 3C). These data support the hypothesis that DNA methylation silences PR, and that this mechanism is most frequently observed in poorly differentiated endometrial cancer cells.

### PR transcriptional repression is reversed by a histone deacetylase inhibitor

**Figure 4:**
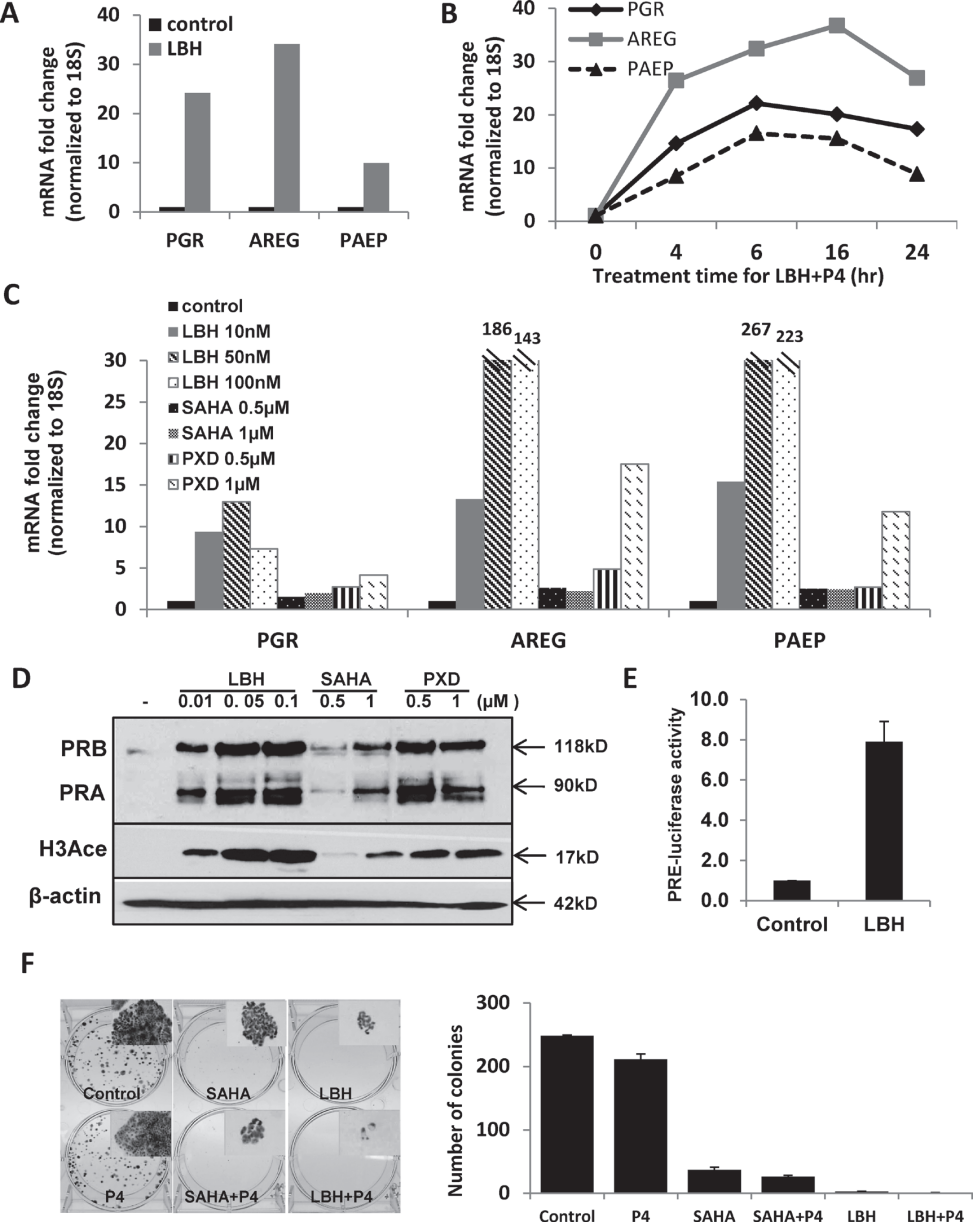
Histone deacetylase (HDAC) inhibition restores PR mRNA and protein expression in Type I Ishikawa H cells **(A)** q-PCR analysis. Ishikawa H cells were treated with DMSO control or 20 nM LBH589 (LBH) for 24 h. *PGR, AREG* and *PAEP* mRNA expression was normalized to 18S, and all q-PCR data are displayed as fold-change relative to DMSO control. Comparisons of normalized expression values (ΔCt) employed the conventional ΔΔCt fold change method. **(B)** PR expression and activity corresponding to time course of P4 stimulation: Ishikawa H cells were treated with 20 nM LBH +100 nM P4 for the indicated times, and *PGR*, *AREG* and *PAEP* mRNA expression was quantified by q-PCR and normalized to 18S. **(C)** q-PCR analysis. Ishikawa H cells were treated with three different HDAC inhibitors (LBH589, SAHA and PXD101) at the indicated concentrations for 24h. *PGR*, *AREG* and *PAEP* mRNA expression was quantified by q-PCR and normalized to 18S. **(D)** Western blotting. Expression of PR protein in Ishikawa H was measured after treating with the three HDAC inhibitors. The presence of histone H3 acetylation indicates drug effect, and β-actin serves as a loading control. **(E)** PRE-luciferase assay. Ishikawa H cells were treated with 20 nM LBH589 for 24h and studied using a PRE-luciferase assay. The PRE-luciferase activity was normalized to total protein concentration. **(F)** Colony formation assay. Ishikawa H cells were treated in the presence or absence of HDACi for 2 weeks, and resulting colonies were stained with crystal violet (left panel, insets are 5X) and the number of colonies recorded (right panel). Error bar, SD.

The lack of promoter methylation in some cell lines suggests that alternative mechanisms mediate PR downregulation in different types of endometrial cancer. Several reports have demonstrated that treatment with a histone deacetylase inhibitor restores PR mRNA and protein expression in endometrial cancer cell lines [18, 19]. Therefore, we studied the effect of the potent histone deacetylase inhibitor (HDACi) LBH589 on PR expression in moderately differentiated Ishikawa H cells [16]. LBH589 restored *PGR* mRNA expression as well as levels of target gene transcripts *AREG* and *PAEP* (Fig. 4A). LBH589 treatment also upregulated *PGR* mRNA expression in KLE endometrial cancer cells (Fig. S2D). To confirm that the increased mRNA expression of *AREG* and *PAEP* was PR-dependent, progesterone was added and expression of these genes was assessed over time. *PGR* and *PAEP* transcripts reached the highest levels at 6 h, while *AREG* mRNA peaked at 16 h. Expression was gradually reduced by 24 h, consistent with PR degradation (Fig 4B). Next, we studied other HDAC inhibitors to confirm the generality of our findings. In addition to LBH589, two other pan-HDACi agents were evaluated, SAHA and PXD101. Similar results were obtained (Fig. 4C), and PR protein expression was induced in addition to message (Fig. 4D). We also detected increased histone H3 acetylation, which confirms the drug effect (Fig. 4D). For less potent HDAC inhibitors, such as SAHA, extending the treatment from 24 to 72 h sustained PR expression at both the message and protein levels and resulted in increased expression of PR target genes *AREG* and *PAEP* in the presence of ligand (Fig. S3). To further verify that the PR induced in response to HDACi treatment is transcriptionally active, a construct containing the progesterone response element (PRE) upstream of a luciferase reporter gene (PRE-luciferase) was transfected into Ishikawa H cells and luciferase activity was monitored. LBH589 treatment increased PRE-luciferase activity 8-fold when compared with the DMSO control (Fig. 4E), thereby confirming the activity of PR. We further explored whether PR induced in response to an HDACi resulted in the expected inhibition of Ishikawa H cell growth as determined by colony formation. Fig. 4F demonstrates that SAHA or LBH589 treatment decreased Ishikawa H cell colony size and number. When progesterone was added in addition to the HDACi, a further reduction in colony size and number resulted. These data demonstrate that PR expression is repressed but can be induced by HDACi treatment in some endometrial cancer cells, and the growth limiting effects of progesterone can be magnified as a result.

### PR transcriptional repression by the polycomb repressor complex 2 (PRC2)

**Figure 5:**
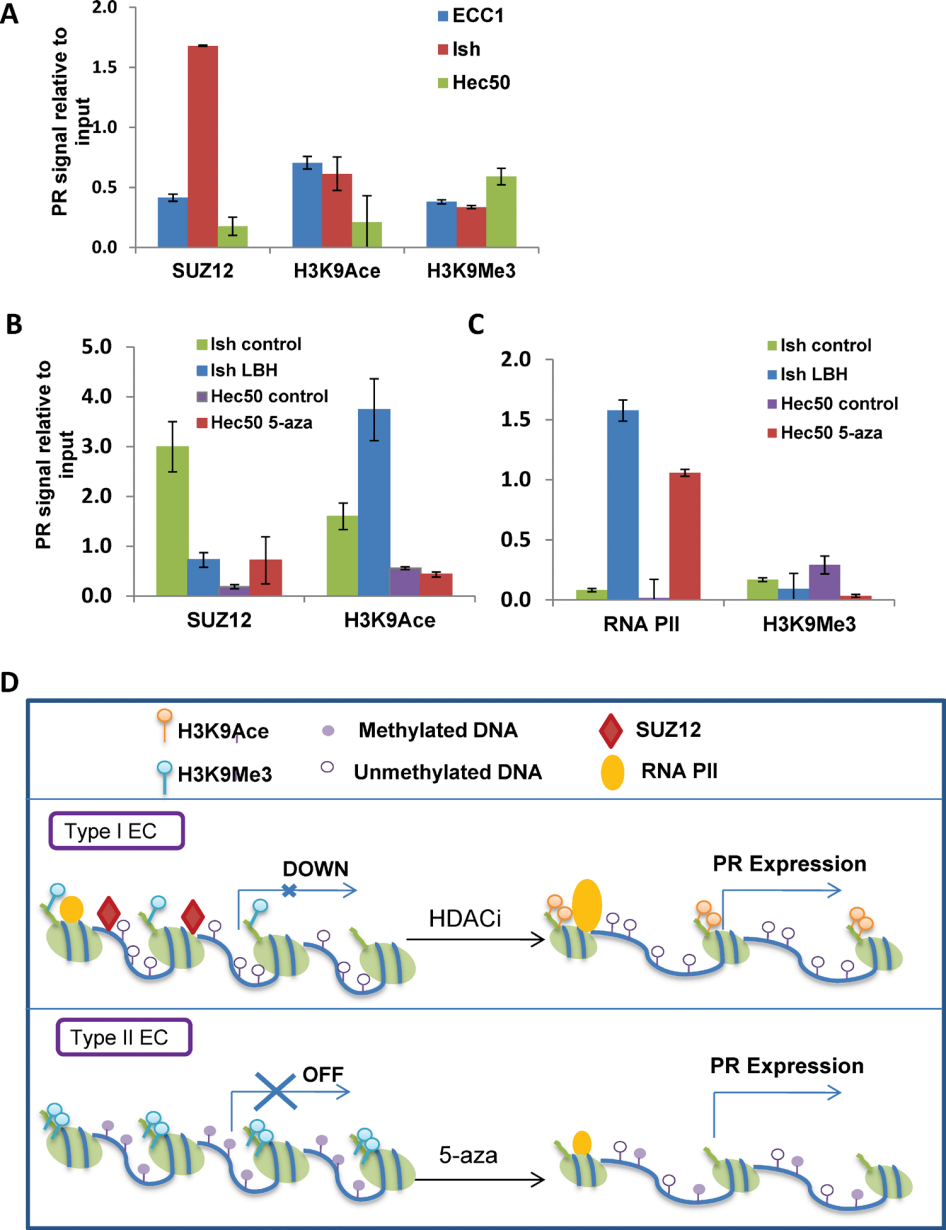
Mechanisms of progressive PR silencing in endometrial cancer cells **(A)** ChIP followed by q-PCR analysis of SUZ12, H3K9Ace or H3K9Me3 recruitment to the *PGR* promoter. **(B)** ChIP-PCR analysis. Ishikawa H cells were treated with or without 20 nM LBH589 for 24h, and Hec50 cells were treated with or without the hypomethylating agent 100 nM 5-aza-deoxycytidine (5-aza) for 5 days. ChIP followed by q-PCR for SUZ12 and H3K9Ace was used to determine recruitment of these factors to the *PGR* promoter. **(C)** ChIP-PCR analysis. ChIP followed by q-PCR for RNA polymerase II (RNA PII) and H3K9 trimethylation (H3K9Me3) was performed to assess occupancy on the *PGR* promoter. **(D)** Proposed model for PR repression in well-differentiated and poorly-differentiated endometrial cancer. In well-differentiated endometrial cancers, PR was transcriptional repressed by PRC2 and reversed by HDACi treatment, while in poorly-differentiated endometrial cancers, PR was suppressed by DNA methylation and reversed by a hypomethylating agent.

We next sought to understand the mechanism of transcriptional repression of PR in Ishikawa H cells which model moderately differentiated endometrial tumors [16]. Two publications have previously reported that the PRC2 binds to the *PGR* promoter and inhibits transcription in breast cancer cells [20, 21]. To investigate this premise in endometrial cancer cells, chromatin immunoprecipitation (ChIP) was performed to determine whether components of the polycomb repressor complex 2, such as SUZ12, bind to the *PGR* promoter in Ishikawa H cells. Well-differentiated ECC1 cells (PR high, Fig. S2B) and poorly differentiated Hec50co cells (PR lowest, Fig. S2B) were used as controls. ChIP data revealed that Ishikawa H cells have the highest percentage of SUZ12 bound to the *PGR* promoter (1.7%) vs. ECC1 (0.4%) and Hec50co (0.2%, Fig. 5A). These data indicate that polycomb repression contributes to the modest PR expression in Ishikawa H cells. Next, we examined histone modifications as markers of PR gene transcriptional status based upon the fact that H3 lysine 9 acetylation (H3K9Ace) indicates active gene transcription, while H3K9 methylation (H3K9Me) is consistent with transcriptional repression [22]. Whereas ECC1 cells had enhanced H3K9Ace, indicative of higher PR expression, Hec50co cells demonstrated the lowest level of H3K9Ace and the highest amount of HeK9Me, consistent with low PR expression in response to gene methylation and confirming our previous findings.

We hypothesized that the mechanism of LBH589-induced PR expression in Ishikawa H cells is the dissociation of PRC2 component SUZ12 from the *PGR* promoter, increased binding of acetylated H3K9 and enhanced RNA polymerase II binding. As shown in Fig. 5B, LBH589 treatment decreased the binding of SUZ12 on the *PGR* promoter from 3% before treatment to 0.8% after treatment. Acetylated H3K9 was increased on the PR promoter, from 1.6% before treatment to 3.7% after treatment. LBH589 treatment also enhanced the binding of RNA polymerase II to the promoter (Fig 5C). By contrast, SUZ12 was not present on the PR promoter in poorly differentiated Hec50co cells, where the promoter is methylated. Consistent with this finding, 5-aza-dC but not LBH589 reversed H3K9 methylation and promoted RNA polymerase II binding to the Hec50co *PGR* promoter (Fig. 5C).

Taken together, our data suggest that there are at least two different mechanisms of PR downregulation which relate to epigenetic modifications. Fig. 5D summarizes these data: in moderately differentiated endometrial cells (commonly referred to as Type I cancer cells), the modest PR expression is due to *PGR* promoter silencing, and SUZ12 bound to the promoter is a marker for this effect. An HDACi can reverse this silencing mechanism. In contrast, the *PGR* promoter in poorly differentiated (Type II) cells is permanently marked by DNA methylation. A hypomethylating agent can partially reverse this effect and boost PR transcription.

### miRNAs reversibly fine-tune PR expression

**Figure 6:**
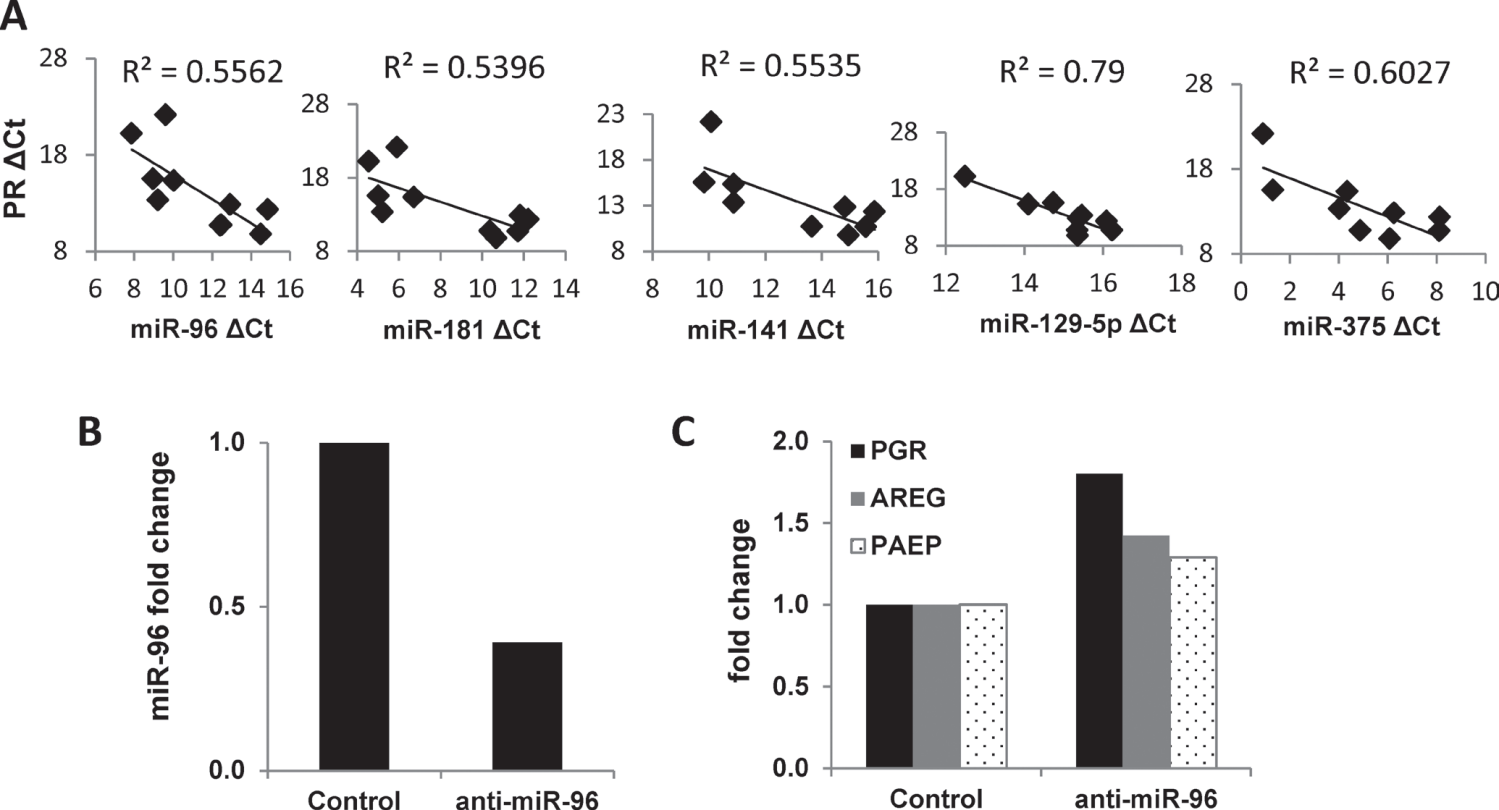
Impact of miRNAs on PR expression in endometrial tumors and cells **(A)** Correlation of miRNA expression with *PGR* expression in matched non-malignant endometrial tissue vs. endometrioid adenocarcinoma (n=5). mRNA expression of *PGR* was measured by q-PCR and normalized to 18S and miRNA expression was measured by q-PCR and normalized to RNU48, and both data are displayed as ΔCt value relative to the DMSO control. **(B)** miR-96 was decreased by transfecting the miR-96 inhibitor into Ishikawa H cells. **(C)** PR expression was restored by inhibiting miR-96 in Ishikawa H cells. Comparisons of normalized miRNA expression values (ΔCt) employed the conventional ΔΔCt fold change method.

miRNAs are a class of small non-coding RNAs that can orchestrate complex posttranscriptional gene expression by various mechanisms, such as mRNA degradation, transcriptional repression and inhibition of translation [23]. Acting in concert, multiple miRNAs fine tune the expression of important factors in endometrial cancer [24]. Several miRNAs are reported to target PR: miR-96 in endometrial cancer and miR-26a and miR-181a in MCF7 breast cancer cells [25, 26]. To further understand the role of miRNAs in PR regulation, de-identified tumor samples from five patients with early stage and grade endometrial cancer were analyzed along with matched adjacent non-malignant tissue. In Figure 1, we established that *PGR* expression is decreased significantly in the tumor tissue compared to the adjacent tissue. Next, we correlated these findings with the expression of miRNAs believed to regulate PR in those samples. We found that the expression of five miRNAs was inversely correlated with PGR expression, among them and most prominently, miR-96 (Fig. 6A). We hypothesize that these miRNAs may mediate PR post-transcriptional repression. To confirm PR as a direct target of these miRNAs, anti-miRNAs were transiently transfected into Ishikawa H cells. Of the tested miRNAs, the most robust effect on PR was achieved with anti-miR-96. Specifically, *PGR* mRNA expression as well as expression of PR target genes *AREG* and *PAEP* was increased (Fig. 6C).

### Integrated analysis of PR downregulation mechanism with endometrial cancer progression

**Figure 7:**
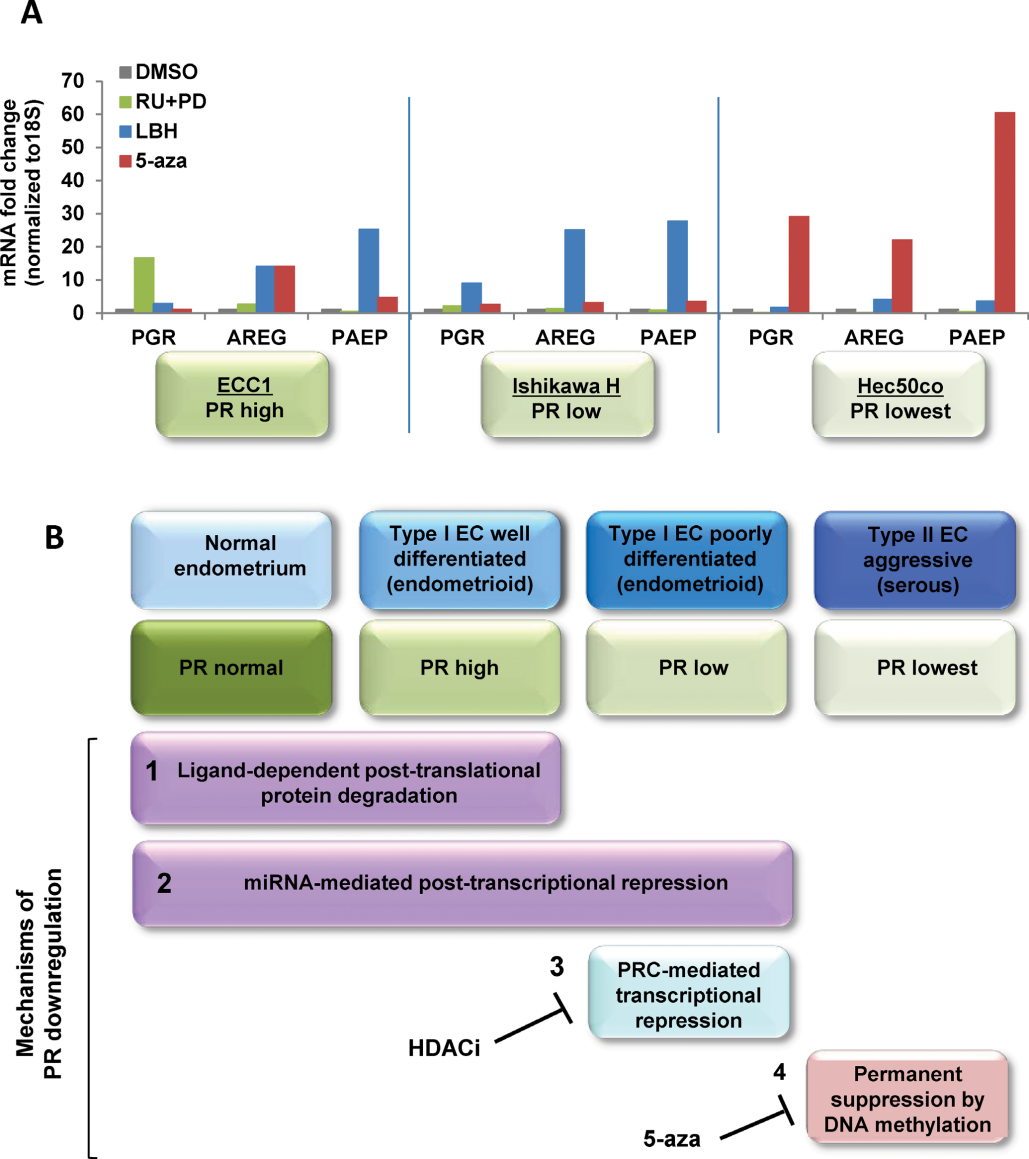
Systematic analysis of strategies to restore functional PR expression in distinct models of endometrial cancer **(A)** Three endometrial cancer model cell lines, ECC1, Ishikawa H and Hec50co cells were treated with DMSO control, 1 μM RU486+1 μM PD0325901 for 24h, 20 nM LBH589 for 24h or 100 nM 5-aza for 3 days. mRNA expression of *PGR*, *AREG* and *PAEP* were measured by q-PCR, normalized to 18S and displayed as fold change to DMSO control. Comparisons of normalized expression values (ΔCt) employed the conventional ΔΔCt fold change method. In ECC1 cells, which express PR at baseline, treatment with the progestin antagonist RU486 and the MAPK inhibitor PD0325901 validates the dynamic PR regulation by ligand-mediated degradation. In Type I Ishikawa cells, only an HDACi effectively restores functional PR, whereas a hypomethylating agent is necessary for Type II Hec50co cells. **(B)** Proposed model of PR downregulation mechanisms during endometrial cancer progression.

We next systematically evaluated the impact of targeted inhibitors chosen to reverse the individual mechanisms underlying PR downregulation (Fig. 7A). Using the cell lines that model hormone-responsive (ECC1), moderately differentiated/low PR (Ishikawa H), and poorly differentiated/no PR (Hec50co) endometrial cancers, we treated cells with DMSO vehicle control, the PR antagonist (RU486) combined with the MAP kinase inhibitor (PD0325901), the HDAC inhibitor (LBH589), or the hypomethylating agent (5-aza-dC). RU486 plus the MAP kinase inhibitor increased *PGR* only in ECC1 cells, but the PR was inactive as a result of the interference with the normal ligand-activated life cycle of PR. LBH589 restored functional PR expression mainly in Ishikawa H cells where PR was silenced due to binding of the PRC2 to the promoter. LBH589-induced increases in *AREG* and *PAEP* mRNA expression in both ECC1 and Ishikawa H cells suggest that a component of promoter silencing may occur in both cell lines. Finally, 5-aza-dC upregulated *PGR* only in Hec50co cells where the PR promoter is silenced by methylation. A model summarizing the proposed mechanisms underlying the progressive loss of PR is presented in Figure 7B.

## DISCUSSION

The study of progestin action in the endometrium has particular importance because the epithelium relies on progesterone to induce cell differentiation and to counter uncontrolled growth. While progestins have been used with great success to reverse endometrial hyperplasia [27, 28], they are not as effective in the treatment of primary endometrial cancer. Progestins as single agents have been used traditionally in the treatment of recurrent or metastatic endometrial adenocarcinoma with overall response rates ranging from only 8% to 55% [29–33]. It is likely that the lower response rates in a subset of endometrial cancer patients relate to either the initial absence of PR in the tumor or are a result of downregulation of PR in cells that were initially PR-positive [13]. Publications from the Gynecologic Oncology Group (GOG), studies 119 and 153, reported attempts to circumvent progestin-mediated PR downregulation with the strategy of combining tamoxifen, as an estrogen surrogate to induce PR, with intermittent progestin treatment [34–36]. While response rates using this strategy reached 33% in advanced endometrial cancer cases, a magnitude of effect which approximates that of chemotherapy, we propose that the effectiveness of progestin therapy can be further optimized using newer molecular strategies to enhance functional PR expression.

Given that progesterone is the ultimate endometrial tumor suppressor and PR is the key molecule through which it acts, the aims of this study were to 1) understand mechanisms of PR loss with endometrial cancer disease progression by tumor subtype; 2) manipulate and restore functional PR expression; and 3) determine strategies to re-sensitize endometrial cancer cells to progestin-based therapy. Our data elucidated at least four distinct regulatory mechanisms that contribute to PR downregulation in distinct types of endometrial cancer (Fig. 7B). The first mechanism is coarse regulation of protein stability by ligand-dependent activation and proteasomal degradation. The second mechanism is fine-tuning by a group of miRNAs at the post-transcriptional level. The third level of regulation is through polycomb repressor complex-mediated transcriptional repression, and the fourth level is complete transcriptional suppression via DNA methylation. While levels 3 and 4 are irreversible in the endogenous state, the application of epigenetic modulators can reverse these mechanisms and thereby restore functional PR expression.

We first established that PR expression is lost in advanced endometrial by investigating PR expression in a set of endometrial tumors of varying stages (Fig. 1A, 1B). Secondly, we confirmed our clinical findings by studying PR expression using the publicly-available endometrial tumor TCGA database (Fig. 1C). We found that PR expression decreased significantly with increasing disease severity. This conclusion is consistent with many previous reports that loss of PR expression correlates with advanced disease [37, 38]. Next, we investigated the mechanisms which contribute to PR loss. Progestin-dependent PR activation and degradation has been well-studied in breast and endometrial cancer cells [12, 13, 39]. We confirmed that ligand-dependent activation and proteasomal degradation results in lower levels of PR; however, this is the result of PR activity, not inactivity, and is accompanied by the expression of PR-dependent genes. This represents the natural turnover of PR in cells responding to progesterone. The lack of *AREG* and *PAEP* induction by progesterone alone may be due to the time point chosen for study (24h), especially since data in Figure 4B demonstrate that induction of PR target genes is transient. Hence, loss of PR in the setting of progestin therapy initially indicates potential PR activity; however, we propose that with ongoing progestin treatment in the absence of new PR transcription, the beneficial effect of hormonal therapy wanes.

We then evaluated the mechanisms for loss of PR transcription. It is well-established that promoter methylation contributes to decreased gene expression, and this is a mechanism of tumor suppressor loss in cancer. We detected significant methylation of the PR gene in models of advanced endometrial cancer, which is consistent with previous reports [15, 18, 19]. By using the hypomethylating agent, 5-aza-dC, we decreased PR promoter methylation and restored functional PR expression in Type II Hec50co cells, consistent with the findings of others [40]. In addition to this agent, new, potentially more active hypomethylating agents are on the horizon [41]. Therefore, using this strategy to restore PR is promising and will be increasingly important as new agents become available.

The lack of promoter methylation in some PR-negative cells suggested that alternative mechanisms of silencing are in place. Several reports have demonstrated that treatment with an HDACi restores *PGR* mRNA and protein expression in endometrial cancer cell lines [18, 19]. In addition, others have demonstrated that the polycomb repressor complex 2 (PRC2), including binding of SUZ12 and EZH2 to the *PGR* promoter, contributes to *PGR* transcriptional repression in breast cancer cells [20, 21]. We confirmed that PRC2 binding to the *PGR* promoter occurs in moderately-differentiated Ishikawa H cells and is reversed with HDACi treatment. By utilizing three pan-HDACi agents, LBH589, SAHA and PXD101, we reproducibly boosted PR expression, induced PR-dependent genes *AREG* and *PAEP* in cultured cells, and inhibited colony formation in cells modeling moderately-differentiated endometrial cancer. These findings are consistent with previous studies indicating that PR inhibits proliferation, invasion and reduces tumor growth in an endometrial xenograft model [42, 43].

miRNAs constitute yet another epigenetic mechanism involved in the control of PR expression. Some miRNAs are regulated by progesterone/PR while others control PR expression and affect progesterone production [44]. For example, miR-181 and miR-26a are predicted to target PR in breast cancer cells [45], and miR-126-3p has been shown to target PR in mouse mammary epithelial cells [46]. In the human endometrium, miR-96 has been reported to silence PR [25]. In this study, we identified five miRNAs (miR-96, miR-182, miR-141, miR-129-5p and miR-375) which negatively correlate with PR expression in endometrial tissues and propose that these miRNAs further fine tune the expression of PR in endometrial cancer cells.

In summary, our data indicate that with the application of epigenetic modulators, *PGR* silencing can be reversed and functional PR expression restored in endometrial cancer cells that have lost hormone responsiveness. It remains to be determined if the mechanisms of *PGR* epigenetic repression occur sequentially and drive tumor progression. In support of this, others have provided evidence that DNA methylation precedes histone modifications and can act to recruit components of the PRC [47]. Conversely, studies in the literature demonstrate that sequential epigenetic silencing occurs first through histone modifications via the PRC, which then results in recruitment of DNA methyl transferases DNMT1 and DNMT3b [20]. Our data favor the latter mechanism given that the poorly differentiated Hec50 cells demonstrated significant *PGR* promoter methylation and barely detectable SUZ12 binding, whereas the well-differentiated Ishikawa cells had negligible *PGR* promoter methylation and strong PRC occupancy of the *PGR* promoter. However, it is also possible that DNA methylation and histone deacetylation are independent events in tumor progression. Confirmation of these mechanisms of PR suppression in a broader collection of endometrial tumor specimens is needed in order to translate these results into the clinic. Nevertheless, our findings set the stage for future clinical trials combining HDACi and hypomethylating agents with progestin as a means to improve the clinical response to hormonal therapy. We propose that molecularly enhanced hormonal therapy for endometrial cancer has the potential to equal or exceed the clinical benefit of chemotherapy with fewer side effects and at a significantly reduced cost.

## METHODS

Details are provided in *Supplementary Methods*.

### Endometrial tumors and cell lines

Endometrial tumor samples and matched adjacent non-malignant tissue were obtained from the University of Iowa Tissue Procurement Core at the University of Iowa Hospitals and Clinics (with Institutional Review Board approval). All tumor specimens were snap-frozen and stored at −80°C. ECC-1 and T47D cells were purchased from ATCC, and Ishikawa H and Hec50co cells were gifts from Dr. Erlio Gurpide (New York University).

### Immunostaining

Endometrial cancer specimens obtained from the Tissue Procurement Core were subjected to immunohistochemical staining for either total PR or PRB as previously described [4]. Immunofluorescent staining for PR in Hec50co endometrial cancer cells was performed as previously described [48] using anti-PR (#8757, Cell Signaling) [17].

### Real-time PCR

Quantitative real-time PCR (qPCR) was performed as previously described [48]. Comparisons of normalized expression values (ΔCt) employed the conventional ΔΔCt fold change method [49, 50].

### Western blotting

Expression of PR, acetylated histone H3 and β-actin were assessed by Western blotting as previously described [48].

### Methylation-specific PCR and sequencing

DNA methylation was determined by bisulfite sequencing as previously described [51].

### Luciferase assay

Cells were transfected with pPR-luc (Signosis) over 24hr, incubated with 20 nM LBH589 for an additional 24hr, and luciferase activity was determined using the Dual-Luciferase Reporter Assay System (Promega). Results are representative of at least three independent experiments performed in triplicate.

### Chromatin immunoprecipitation assay

Chromatin immunoprecipitation (ChIP) assay was conducted using the SimpleChIP Enzymatic Chromatin IP Kit (Cell Signaling). Results are representative of at least three independent experiments.

### Colony formation assay

Colony formation was determined by counting the number of colonies after 2 weeks in culture. Data are presented as the average number of colonies per well.

### miRNA expression

miRNA-specific qPCR assays for miR-96, miR-182, miR-141, miR-129-5p, and miR-375 (Applied Biosystems) were carried out as previously described [52] on an miRNA panel composed of endometrial endometrioid adenocarcinomas and matched adjacent non-malignant tissue. Data were normalized to RNU48 endogenous RNA control [52]. Ishikawa H cells were transfected with anti-miR96 inhibitor (Life Technology) using Lipofectamine RNAiMAX (Invitrogen). Total RNA was extracted 48 hours after transfection and miRNA and mRNA expression was measured using q-PCR. Comparisons of miRNA normalized expression values (ΔCt) employed the conventional ΔΔCt fold change method [49, 50].

### TCGA data analysis

Patient information was downloaded from The Cancer Genome Atlas Data Portal maintained by National Cancer Institute and National Human Genome Research Institute. Gene expression was assayed based on mRNA sequencing conducted on the Illumina platform and was downloaded from NCI's Cancer Genomics Hub (CGHub). The calculated expression was for all reads aligning to a particular gene per sample. Total of 361 endometrial cancer patients are eligible for *PGR* gene expression analysis. Patients were divided into four groups: endometrioid type I grade 1, grade 2, grade 3 and serous grade 3 which includes cases designated as high grade and mixed histology type. One Way ANOVA was used to detect a significant difference between the groups and the Holm-Sidak method was used for pairwise comparisons. Significance was set as p ≤ 0.05.

### Statistical analyses

Student's t-test was used for comparisons of two groups. All pairwise multiple comparisons were performed by one-way ANOVA using the Holm-Sidak method or Bonferroni post-hoc tests with the overall significance level at 0.05 (p ≤ 0.05).

## SUPPLEMENTARY MATERIALS


