# Supplementary material for: Systematic dissection of the mechanisms underlying progesterone receptor downregulation in endometrial cancer
Source: Oncotarget. 2014 Sep 3;5(20):9783–97. doi: 10.18632/oncotarget.2392 (PMC4259437; doi:10.18632/oncotarget.2392)
Supplement: Supplementary file 1 [file oncotarget-05-9783-s001.pdf]

# SUPPLEMENTARY MATERIALS

## Systematic Dissection of the Mechanisms Underlying Progesterone Receptor Downregulation in Endometrial Cancer

Shujie Yang, Kimberly K. Leslie, Yichen Jia, Xiaoyue Liu, Christopher Winters, Xinjun Wang, Yuping Zhang, Eric J. Devor, Adriann Hovey, Henry D. Reyes, Xue Xiao, Yang Xu, Donghai Dai, Xiangbing Meng, Kristina W. Thiel, Frederick E. Domann

# SUPPLEMENTARY METHODS

## Antibodies and Reagents

5-aza-2'-deoxycytidine (5-aza-dC), progesterone, and RU486 were obtained from Sigma Aldrich. LBH589, SAHA, MS275, PXD101, and PD0325901 were purchased from Selleck Chemicals. All drugs were resuspended in DMSO, except for progesterone and RU486 (ethanol).

Antibodies against PRA/B (#3153), PRB (#3157), and SUZ12 (#3737) were from Cell Signaling. Acetylated histone H3 (H3Ace, #39139), acetylated H3 at lysine 9 (H3K9Ac, #39137), tri-methylated H3 at lysine 9 (H3K9Me3, #39161), and RNA polymerase II (#39097) were from Active Motif. PR (sc-539) and amphiregulin (sc-74501) antibodies were from Santa Cruz Biotechnology. 5-aza-2'-deoxycytidine (5-aza-dC), progesterone, RU486 and β-actin antibody (#A1978) were obtained from Sigma Aldrich.

## Cell lines

Endometrial cancer cell lines AN3CA, RL95-2, Hec1A, SKUT1B, ECC-1, and KLE were purchased from ATCC and grown according to the recommended guidelines. Ishikawa H and Hec50co endometrial cancer cell lines (gifts from Dr. Erlio Gurpide, New York University) were grown in DMEM media supplemented with 10% fetal bovine serum (regular FBS, r-FBS) or charcoal-stripped FBS (cs-FBS, Life Technologies, #12676-011) and penicillin-streptomycin (Gibco).

## Endometrial tumor immunohistochemistry

Endometrial cancer specimens were obtained from the Tissue Procurement Core. Immunohistochemical staining was conducted as previously described [1] using 1:100 dilutions of PR (M3569, DAKO) and PRB (#3157, Cell Signaling) antibodies.

## Real-time PCR

Total RNA was extracted from cultured cells using the miRvana miRNA Isolation kit (Ambion, Life Technologies). RNA yield and purity was assessed using a NanoDrop Model 1000 spectrophotometer (Thermo Scientific). Total RNA (500 ng) was oligo-dT reverse transcribed with SuperScript III (Invitrogen, Life Technologies). Real-time PCR was performed in triplicate on an Applied Biosystems Model 7900 Genetic Analyzer under standard conditions using the following primer sequences: progesterone receptor PGR (PRA/B)-FOR: 5'- ATGTGGCAGATCCCACAGGAGTTT -3' and PGR (PRA/B)-REV: 5'-ACTGGGTTTGACTTCGTAGCCCTT-3', Amphiregulin (AREG), AREG-F: 5'-GTGGTGCTGTCGCTCTTGATA-3' and AREG-R: 5'-CCCCAGAAAATGGTTCACGCT-3', Glycodelin (PAEP), PAEP-F: 5'- GAGATCGTTCTGCACAGATGG -3' and PAEP-R 5'-CGTTCGCCACCGTATAGTTGAT-3'. Note that the *PGR* primers amplify a region conserved in PRA, PRB and PRC. Results were quantitated using the comparative cycle threshold (ΔΔCt) method [2, 3]. 18S rRNA was measured in the same samples (18SFOR: 5'-AACTTTCGATGGTAGTCGCCG-3' (NR_003286, pos.363-383) AND 18SREV: 5'-CCTTGGATGTGGTAGCCGTTT-3' (NR_003286, pos.467-447) and used to correct for variations in RNA content among samples.

## Western blotting

Following treatment, cells were solubilized in cold NP-40 cell lysis buffer (150 mM NaCl, 50 mM Tris/HCl, pH 7.4, 1% NP-40 with a protease and phosphatase inhibitor cocktail from Pierce) and then sonicated to release nuclear proteins as previously described [4]. Lysates were analyzed by Western blotting with specific primary and HRP-conjugated secondary antibodies. For detection of PR protein, a combination of the PRA/B (#3153, Cell Signaling) and PRB (#3157, Cell Signaling) antibodies at a 1:1000 dilution each was used.

## Immunostaining

Cells were grown on glass coverslips and treated with indicated reagents. After fixation with 2% paraformaldehyde and permeabilization with 1% Triton X-100, cells were labeled with a PR antibody (#8757, Cell Signaling) as described [5] followed by Alexa Fluor-488 conjugated secondary antibody. Images were visualized by fluorescence microscopy and acquired with an Olympus BX51 camera.

## Methylation-specific PCR and sequencing

DNA methylation was assessed by bisulfite sequencing as previously described [6]. Briefly, genomic DNA (gDNA) was prepared from cultured cells using the DNeasy kit according to manufacturer's recommendations (Qiagen). Yield and purity of the gDNA was verified using a NanoDrop Model 1000 spectrophotometer (Thermo Scientific). Bisulfite conversion of 2µg aliquots of gDNA from each cell line was carried out using the EZ DNA Methylation Direct kit according to manufacturer's recommendations (ZYMO Research).

Bisulfite-converted DNA was PCR amplified using primer sequences flanking a CpG island just upstream of the PGR start codon. Primers were designed to specifically amplify bisulfite converted DNA (bisDNA). Primer sequences: PGRbisFOR: 5'-ATTAGTTTTAGGGTT

TTAGTGAGGG-3' (NM_000926, pos.228-252) and PGRbisREV: 5'- CTCCAAAAAAATTC

TCCAACTTCTATC-3' (NM_000926, pos.440-414). PCR amplicons were cloned into pGEM T-EASY (Promega) and multiple clones were sequenced on an Applied Biosystems Model 3730xl DNA sequencer. Multiple clones were sequenced in order to detect methylation heterogeneity, if any, in the eleven CpG dimers in the amplicons.

## Luciferase assay

Ishikawa cells ($5x10^5$) were plated in 6-well plates and transfected with a reporter plasmid, pPR-luc (Signosis), that contains 3 progesterone response elements in tandem. Transfections were performed with FuGene 6 (Roche). After 24 hrs, cells were incubated with 20 nM LBH589 or DMSO control for an additional 24 hr. Luciferase activities were measured using a Dual-Luciferase Reporter Assay System (Promega) and a microplate luminometer (Infinite M200, TECAN). Data were normalized to total protein concentration as determined based on the method of Bradford (Bio-Rad). Results are representative of at least 3 independent experiments. All experiments were performed in triplicate.

## Chromatin immunoprecipitation assay

Chromatin immunoprecipitation (CHIP) assay was conducted using SimpleChIP Enzymatic Chromatin IP Kit (magnetic beads, #9003, Cell Signaling) according to the manufacturer's protocol. After treatment as indicated, cells were cross-linked with paraformaldehyde, digested with micrococcal nuclease to achieve a DNA fragment of 200-1000 bp, and incubated with antibodies against SUZ12 (#3737, Cell Signaling), acetylated H3 at lysine 9 (H3K9Ac, #39137), tri-methylated H3 at lysine 9 (H3K9Me3, #39161), and RNA polymerase II (#39097), all were from Active Motif per manufacturer's instructions (SimpleChIP Enzymatic Chromatin IP Kit). Next, qRT-PCR was performed in triplicate using 3µl of DNA extract with the following primers: PGR promoter (PRB) 5'- GAGGAGGAGGCGTTGTTAGA -3' (forward) and 5'- GCCTCGGGTTGTAGATTTCA -3' (reverse) and POWER SYBR Green PCR Master Mix (Applied Biosystems, #4367659) on the Applied Biosystems Model 7900 Genetic Analyzer under standard conditions. Data were quantitated relative to the input (fragmented DNA). Results are representative of at least 3 independent experiments.

## Colony formation assay

Ishikawa cells (750 cells) were seeded in triplicate in 6 well plates. Cells were treated with DMSO vehicle, 1 µM SAHA or 10 nM LBH589 with or without P4 (100 nM) every other day for 2 weeks. Colonies were stained with crystal violet for image acquisition. Data are presented as the average number of colonies per well.

## miRNA expression

miRNA-specific qPCR assays for miR-96, miR-182, miR-141, miR-129-5p, and miR-375 were carried out using miRNA-specific RT primers and qPCR primer/probe sets (Life technology, Applied Biosystems) on an RNA panel composed of the matched adjacent non-malignant tissue and endometrial endometrioid adenocarcinomas tissue as previously described [7]. These assays were run on an Applied Biosystems Model 7900 Genetic Analyzer and the resulting data again were analyzed using the Applied Biosystems StatMiner software following normalization against the RNU48 endogenous RNA control [7].

Ishikawa H cells were transfected with anti-miR96 inhibitor (Life Technology) using Lipofectamine RNAiMAX (Invitrogen). Total RNA was extracted 48 hours after transfection and miRNA and mRNA expression was measured using q-PCR. Comparisons of miRNA normalized expression values ($\Delta$Ct) employed the conventional $\Delta\Delta$Ct fold change method [2, 3].

## TCGA data analysis

Patient information was downloaded from The Cancer Genome Atlas Data Portal maintained by National Cancer Institute and National Human Genome Research Institute. Gene expression was assayed based on mRNA sequencing conducted on the Illumina platform and was downloaded from NCI's

Cancer Genomics Hub (CGHub). The calculated expression was for all reads aligning to a particular gene per sample. There were a total of 361 endometrial cancer patients eligible for progesterone receptor gene expression analysis. Patients were divided into four groups: endometrioid grade 1, endometrioid grade 2, endometrioid grade 3 and serous grade 3 which includes cases designated as high grade and mixed histology type. One Way ANOVA was used to detect a significant difference between the groups and the Holm-Sidak method was used for pairwise comparisons. Significance was set as $p \leq 0.05$.

### Statistical analyses

Student's t-test was used for comparisons of two groups. All pairwise multiple comparisons were performed by one-way ANOVA using the Holm-Sidak method or Bonferroni post-hoc tests with the overall significance level at 0.05 ($p \leq 0.05$).

## SUPPLEMENTARY TABLE AND FIGURES

**Supplementary Table S1: Primers for real-time PCR and sequencing of *PGR methylation*.**

| Primer name | Forward primer | Reverse primer |
|---|---|---|
| PGR | ATGTGGCAGATCCCACAGGAGTTT | ACTGGGTTTGACTTCGTAGCCCTT |
| AREG | GTGGTGCTGTCGCTCTTGATA | CCCCAGAAAATGGTTCACGCT |
| PAEP | GAGATCGTTCTGCACAGATGG | CGTTCGCCACCGTATAGTTGAT |
| 18S | AACTTTCGATGGTAGTCGCCG | CCTTGGATGTGGTAGCCGTTT |
| PGRbis | ATTAGTTTTAGGGTTTTAGTGAGGG | CTCCAAAAAAATTCTCCAACTTCTATC |
| PGR(B)-ChIP | GAGGAGGAGGCGTTGTTAGA | GCCTCGGGTTGTAGATTTCA |

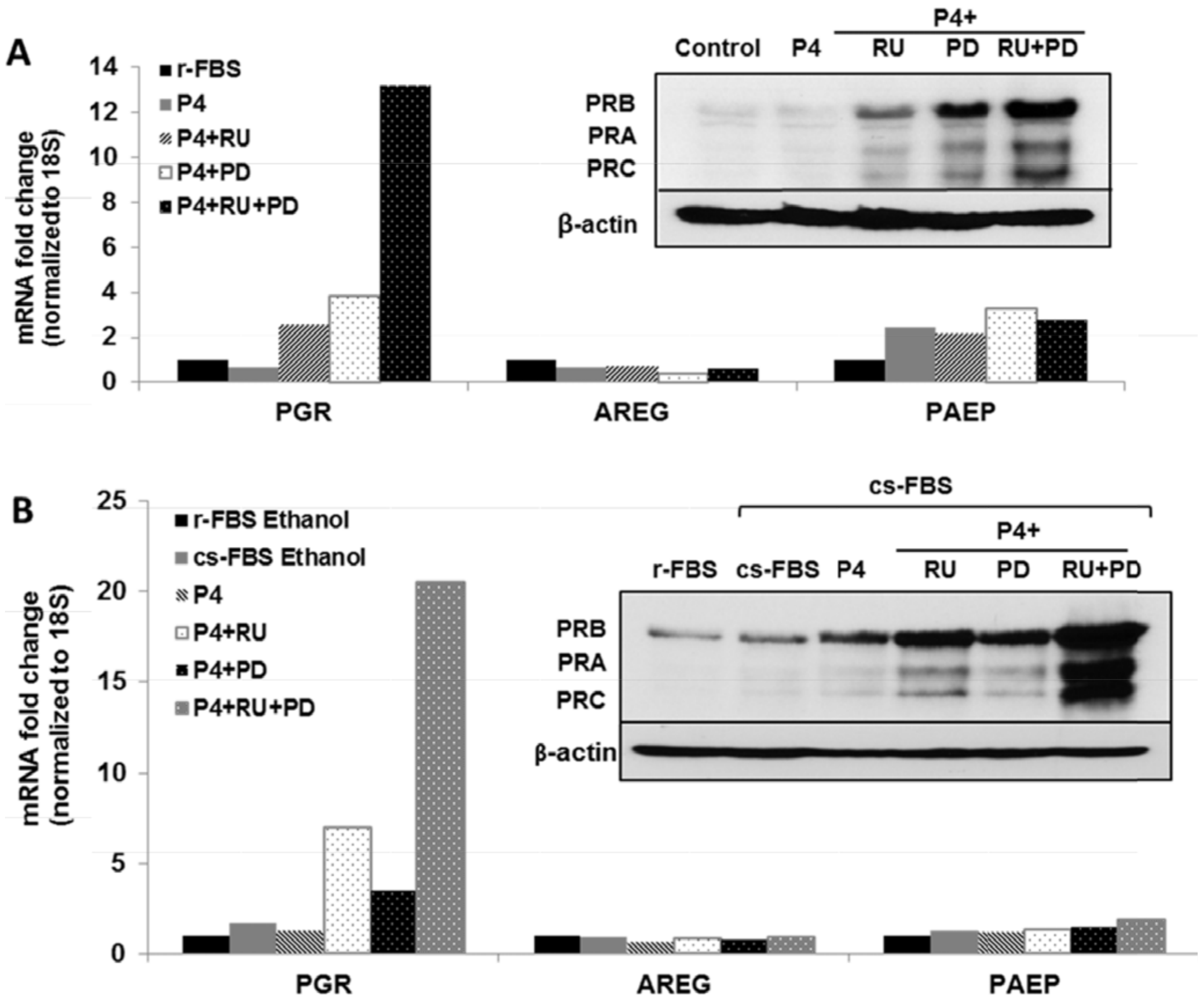


**Supplementary Figure S1: Ligand-dependent PR downregulation in response to control versus stripped fetal bovine serum (FBS).** q-PCR and Western blotting: after ECC1 cells were grown in RPMI1640 media supplemented with regular FBS (r-FBS) (**A**) or charcoal-stripped FBS (cs-FBS) (**B**) for 24 h, ECC1 cells were treated with DMSO vehicle control, 100 nM P4, 100 nM P4 and 1 µM of the PR antagonist RU486 (RU), 100 nM P4+ 1 µM of the MAPK inhibitor PD0325901 (PD) or the combination of P4+RU+PD for an additional 24h. mRNA expression of *PGR* was measured by q-PCR, normalized to 18S, and data are displayed as fold-change relative to the DMSO control. The insert demonstrates PR protein expression after the same treatments; β-actin, loading control.

**A** Methylated CpG ratio (% methylation)

| Methylation position | KLE | AN3CA | SKUT1B | ECC1 | Ishikawa | RL95 | Hec1A | Hec50co |
|---|---|---|---|---|---|---|---|---|
| -332 | 0/11 (0%) | 1/11 (9%) | 0/11 (0%) | 0/11 (0%) | 1/11 (9%) | 9/11 (82%) | 11/11 (100%) | 5/12 (42%) |
| -345 | 0/11 (0%) | 0/11 (0%) | 1/11 (9%) | 0/11 (0%) | 0/11 (0%) | 10/11 (91%) | 11/11 (100%) | 3/12 (25%) |
| -364 | 0/11 (0%) | 0/11 (0%) | 1/11 (9%) | 0/11 (0%) | 0/11 (0%) | 7/11 (64%) | 11/11 (100%) | 12/12 (100%) |
| -381 | 0/11 (0%) | 0/11 (0%) | 1/11 (9%) | 1/11 (9%) | 0/11 (0%) | 10/11 (91%) | 11/11 (100%) | 12/12 (100%) |
| -387 | 0/11 (0%) | 0/11 (0%) | 0/11 (0%) | 0/11 (0%) | 0/11 (0%) | 10/11 (91%) | 11/11 (100%) | 12/12 (100%) |
| -390 | 0/11 (0%) | 1/11 (9%) | 0/11 (0%) | 0/11 (0%) | 0/11 (0%) | 10/11 (91%) | 11/11 (100%) | 12/12 (100%) |
| -406 | 0/11 (0%) | 0/11 (0%) | 0/11 (0%) | 0/11 (0%) | 0/11 (0%) | 6/11 (55%) | 11/11 (100%) | 12/12 (100%) |
| -408 | 0/11 (0%) | 0/11 (0%) | 0/11 (0%) | 0/11 (0%) | 0/11 (0%) | 7/11 (64%) | 11/11 (100%) | 12/12 (100%) |
| -424 | 0/11 (0%) | 0/11 (0%) | 0/11 (0%) | 0/11 (0%) | 0/11 (0%) | 9/11 (82%) | 11/11 (100%) | 12/12 (100%) |
| -448 | 0/11 (0%) | 0/11 (0%) | 0/11 (0%) | 0/11 (0%) | 0/11 (0%) | 2/11 (18%) | 0/11 (0%) | 12/12 (100%) |
| -476 | 0/11 (0%) | 0/11 (0%) | 0/11 (0%) | 0/11 (0%) | 0/11 (0%) | 3/11 (27%) | 0/11 (0%) | 11/12 (92%) |
| Overall methylation | 0/121 (0%) | 2/121 (2%) | 3/121 (2%) | 1/121 (1%) | 1/121 (1%) | 83/121 (69%) | 99/121 (82%) | 115/132 (87%) |

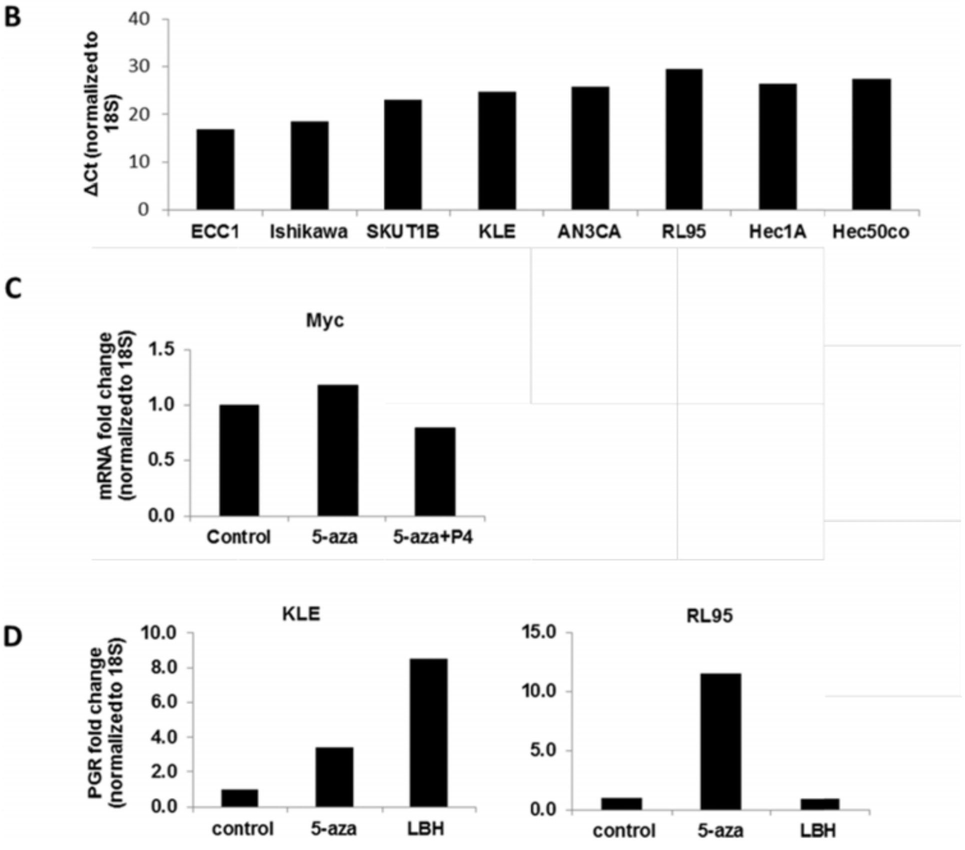


**Supplementary Figure S2: 5-aza-dC treatment induces PR expression in endometrial cancer cells with a highly methylated *PGR* promoter. (A)** PR promoter methylation in 8 endometrial cancer cell lines. **(B)** PR mRNA expression in 8 endometrial cancer cell lines. **(C)** mRNA expression of the oncogene Myc was unchanged following treatment with 5-aza-dC. **(D)** The HDACi, LBH589, restores PR expression in KLE cells (left panel). 5-aza-dC enhanced PR expression in RL95 cells with a hyper-methylated PR promoter, but not in KLE cells which lack PR promoter methylation (right panel).

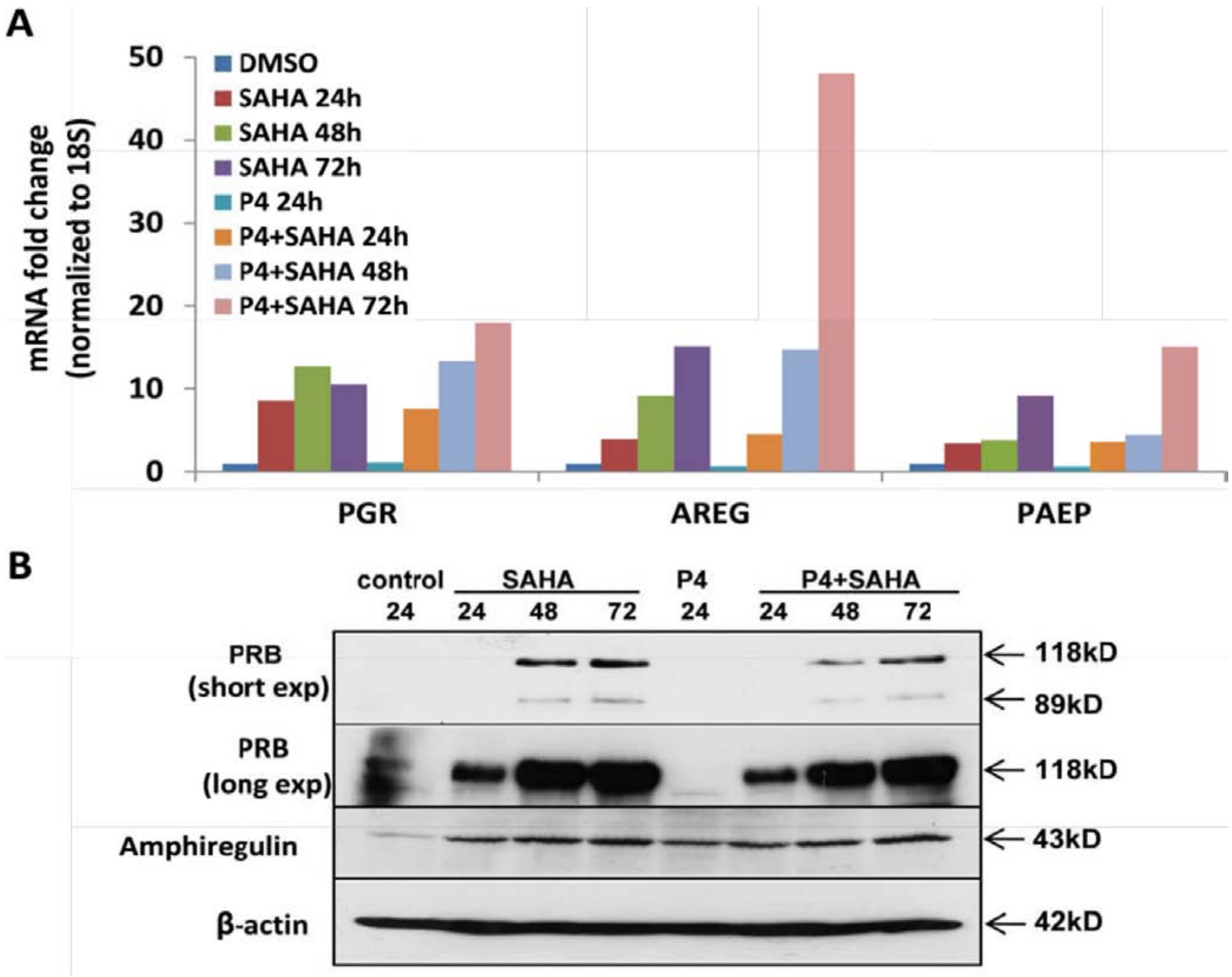


**Supplementary Figure S3: The HDACi SAHA increases functional PR mRNA and protein expression. (A)** Induction of PGR, AREG and PAEP in response to the indicated regimens in Ishikawa H cells. **(B)** Induction of PR protein in Ishikawa H cells in response to the indicated treatment regimens.

## SUPPLEMENTARY REFERENCES

1. Singh M, Zaino RJ, Filiaci VJ and Leslie KK. Relationship of estrogen and progesterone receptors to clinical outcome in metastatic endometrial carcinoma: A Gynecologic Oncology Group Study. Gynecol Oncol. 2007; 106(2):325–333.
2. Livak KJ and Schmittgen TD. Analysis of relative gene expression data using real-time quantitative PCR and the 2(-Delta Delta C(T)) Method. Methods. 2001; 25(4):402–408.
3. Schmittgen TD and Livak KJ. Analyzing real-time PCR data by the comparative C(T) method. Nat Protoc. 2008; 3(6):1101–1108.
4. Yang S, Xiao X, Jia Y, Liu X, Zhang Y, Devor EJ, Meng X, Thiel KW and Leslie KK. Epigenetic Modification Restores Functional PR Expression in Endometrial Cancer Cells. Curr Pharm Des. 2013.
5. Cheung WC, Beausoleil SA, Zhang X, Sato S, Schieferl SM, Wieler JS, Beaudet JG, Ramenani RK, Popova L, Comb MJ, Rush J and Polakiewicz RD. A proteomics approach for the identification and cloning of monoclonal antibodies from serum. Nat Biotechnol. 2012; 30(5):447–452.
6. Rose SL, Fitzgerald MP, White NO, Hitchler MJ, Futscher BW, De Geest K and Domann FE. Epigenetic regulation of maspin expression in human ovarian carcinoma cells. Gynecol Oncol. 2006; 102(2):319–324.
7. Devor EJ, Hovey AM, Goodheart MJ, Ramachandran S and Leslie KK. microRNA expression profiling of endometrial endometrioid adenocarcinomas and serous adenocarcinomas reveals profiles containing shared, unique and differentiating groups of microRNAs. Oncol Rep. 2011; 26(4):995–1002.
